# Deployment and validation of an AI system for detecting abnormal chest radiographs in clinical settings

**DOI:** 10.3389/fdgth.2022.890759

**Published:** 2022-07-27

**Authors:** Ngoc Huy Nguyen, Ha Quy Nguyen, Nghia Trung Nguyen, Thang Viet Nguyen, Hieu Huy Pham, Tuan Ngoc-Minh Nguyen

**Affiliations:** ^1^Phu Tho Department of Health, Viet Tri, Vietnam; ^2^Medical Imaging Center, Vingroup Big Data Institute, Hanoi, Vietnam; ^3^Smart Health Center, VinBigData JSC, Hanoi, Vietnam; ^4^College of Engineering and Computer Science, VinUniversity, Hanoi, Vietnam; ^5^VinUni-Illinois Smart Health Center, VinUniversity, Hanoi, Vietnam; ^6^Training and Direction of Healthcare Activities Center, Phu Tho General Hospital, Thu Tho, Vietnam

**Keywords:** Computer-Aided Diagnosis, deep learning, clinical validation, Picture Archiving and Communication System (PACS), chest X-ray (CXR)

## Abstract

**Background:**

The purpose of this paper is to demonstrate a mechanism for deploying and validating an AI-based system for detecting abnormalities on chest X-ray scans at the Phu Tho General Hospital, Vietnam. We aim to investigate the performance of the system in real-world clinical settings and compare its effectiveness to the in-lab performance.

**Method:**

The AI system was directly integrated into the Hospital's Picture Archiving and Communication System (PACS) after being trained on a fixed annotated dataset from other sources. The system's performance was prospectively measured by matching and comparing the AI results with the radiology reports of 6,285 chest X-ray examinations extracted from the Hospital Information System (HIS) over the last 2 months of 2020. The normal/abnormal status of a radiology report was determined by a set of rules and served as the ground truth.

**Results:**

Our system achieves an F1 score—the harmonic average of the recall and the precision—of 0.653 (95% CI 0.635, 0.671) for detecting any abnormalities on chest X-rays. This corresponds to an accuracy of 79.6%, a sensitivity of 68.6%, and a specificity of 83.9%.

**Conclusions:**

Computer-Aided Diagnosis (CAD) systems for chest radiographs using artificial intelligence (AI) have recently shown great potential as a second opinion for radiologists. However, the performances of such systems were mostly evaluated on a fixed dataset in a retrospective manner and, thus, far from the real performances in clinical practice. Despite a significant drop from the in-lab performance, our result establishes a reasonable level of confidence in applying such a system in real-life situations.

## 1. Introduction

Chest radiograph, or chest X-ray (CXR), remains one of the most common, yet hard to interpret, imaging protocols in medicine. It is hoped that a Computer-Aided Diagnosis (CAD) system using artificial intelligence (AI) can effectively assist radiologists and help mitigate the misdiagnosis rate on CXRs. Leveraging recent advances in deep learning (1), such systems have achieved a great success in detecting a wide range of abnormalities on CXRs (2–13). Most of the existing systems are supervised-learning models trained and validated on different parts of a dataset that was collected and labeled in a retrospective fashion. For example, several deep learning models were developed (3, 5) on the ChestX-ray14 dataset (14) for classifying 14 common thoracic pathologies. Recently, most algorithms for detecting abnormalities on CXRs were trained and validated on the CheXpert (4, 6, 12) and MIMIC-CXR (15) datasets, which include the same set of 14 findings that are slightly different from the labels of ChestX-ray14. The performances of the aforementioned AI systems in differentiating multiple findings on CXRs were reported to be comparable with radiologists. Other works were devoted to detecting a specific lung disease such as pneumonia (2), pulmonary tuberculosis (9, 11) and lung cancer (16). Notably, Rajpurkar et al. (2) trained a convolutional neural network (CNN) for detecting pneumonia that achieved an F1 score of 0.435 (95% CI 0.387, 0.481) on the ChestX-ray14 dataset, which performance was claimed to exceed those of practicing radiologists. Tang et al. (17) proposed to train an abnormality classifier, which is closely related to our work, with various CNN architectures over three CXR different datasets: the ChestX-ray14, the RSNA Pneumonia Detection Challenge (18), and the Indiana University Hospital Network (19). Although reaching impressive AUC (Area under the receiver operating characteristic Curve) performances of 0.9x, those models were again evaluated on retrospective curated datasets that might be drastically different from the real data in clinical settings.

Unlike existing works, our study does not focus on developing and retrospectively evaluating an AI-based CAD system for CXR. Instead, we propose a framework to prospectively validate such a system while deployed at a clinical site for a significantly long period. In particular, we integrate our system, VinDr-CXR, directly to the Picture Archiving and Communication System (PACS) of the Phu Tho General Hospital—a provincial hospital in Vietnam. The system consists of three AI models that were trained on our dataset (20) collected from other sources. All CXRs generated by the PACS during 2 months are prospectively automatically analyzed by the VinDr-CXR. The obtained AI results are then matched and compared with the radiology reports extracted from the Hospital Information System (HIS) to compute the system's performance in distinguishing abnormal vs. normal CXR studies. Despite the ability of the system to localize multiple classes of lesions, we only measure its performance as a binary classifier. The reason for doing so is that it is much more reliable to decide if a radiology report is abnormal than to interpret its subtle details. We also propose simple template matching rules to determine the normal/abnormal status.

Over the last 2 months of 2020, the VinDr-CXR system generated AI results for 6,687 CXR studies taken at the Phu Tho General Hospital, 6,285 of which were matched with corresponding radiology reports from the HIS. The matching was nontrivial since the PACS, and the HIS were not linked by accession numbers. Instead, we had to rely on the patient ID, and other attributes of the Digital Imaging and Communications in Medicine (DICOM) files and the radiology reports. By treating the normal/abnormal status of the 6,285 matched reports as a ground-truth reference, the abnormality classifier of the VinDr-CXR yielded an F1 score of 0.653 [95% Confidence Interval (CI) 0.635, 0.671]. The 95% CI of the F1 score statistic was obtained by bootstrapping (21), a method that was also used in the work of Rajpukar et al. (2). The F1 score obtained in this clinical setting is significantly below the one achieved while training and validating the model “at home” on a retrospective dataset. Nonetheless, the reported performance still gives us a high level of confidence in deploying the VinDr-CXR system in clinical practice. It also serves as a good baseline for similar AI-based CAD systems to be clinically validated.

## 2. Materials and methods

The ethical clearance of this study was approved by the Institutional Review Board (IRB) of the Phu Tho General Hospital before the study started. The need for obtaining informed patient consent was waived because this study did not impact clinical care or workflow at the hospital, and all patient-identifiable information in the data has been removed.

**Figure 1:**
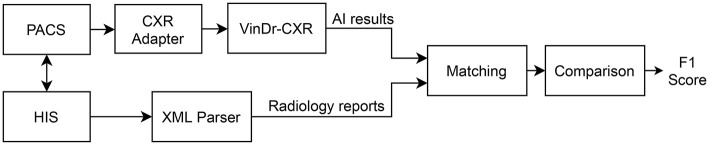
Validation scheme. PACS and HIS are linked by Patient ID.

We propose an overall scheme for validating our CAD system, VinDr-CXR, as illustrated in Figure 1. A set of AI results is obtained by directly integrating VinDr-CXR into the Phu Tho General Hospital PACS, while the corresponding radiology reports are extracted from the HIS *via* an Extensible Markup Language (XML) parser. These two sets of results are then pairwise matched and compared to each other to determine the correctness of the system in detecting abnormal CXRs. The final result of the validation will be reported as an F1 score, a metric that balances the precision and recall of a binary classifier.

### 2.1. Development of AI models

**Figure 2:**
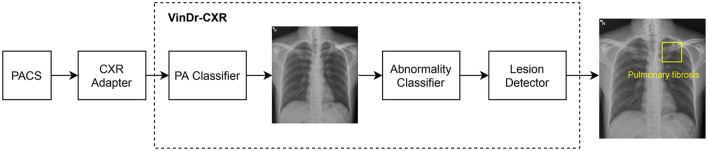
VinDr-CXR pipeline. The system includes three concatenated AI models that are integrated to the PACS *via* a CXR adapter. The output of the system is the probability of the CXR being abnormal and the locations of the lesions, if any.

As shown in Figure 2, the VinDr-CXR system is a concatenation of three AI models: the PA classifier, the abnormality classifier, and, finally, the lesion detector. This system takes as input a CXR from the PACS and returns the probability that the image is abnormal and the locations of multiple classes of lesions if any. All constituent models of VinDr-CXR were obtained by training deep neural networks entirely on our dataset, also called VinDr-CXR, part of which was made publicly available (20). This dataset was retrospectively collected from our partner hospitals in Vietnam and annotated by a team of experienced radiologists. At least one radiologist manually labeled each image in the dataset with a list of six different diagnoses where 22 types of lesions were annotated with bounding boxes. It is important to emphasize that none of the training data was from the Phu Tho General Hospital. Each model was trained and validated before being deployed in the real clinical workflow of the hospital. We did not make any changes to the models during the 2 months of the clinical trial. This is to ensure that our models are not biased to the real-life validation setup. We briefly describe the development of the three AI models; details of the training will be presented somewhere else.

#### 2.1.1. PA classifier

This PA classifier is attached to the CXR adapter to guarantee that only CXRs of the Posterior-Anterior (PA) view will be passed to the abnormality classifier, trained only on this type of image. The output of the PA classifier is a probability of the input image being a PA-view CXR. If this probability is greater than a normalized threshold of 0.5, the image will go through to the abnormality classifier; otherwise, the system will output an indicator that the image is invalid. The PA classifier adopted the ResNet-18 architecture (22) that was trained and validated on a dataset of a total of 9,864 scans, where 4,329 of them are actual PA-view CXRs taken from the VinDr-CXR dataset. The negative training examples included lateral-view CXRs and images of other body parts that sometimes got through the CXR filter due to mismatched DICOM tags. The trained PA classifier achieves an F1 score of 0.980 on a validation set of 4,192 images. Here, the F1 score metric is defined as


(1) $$\begin{array}{r} F_{1}=\frac{TP}{TP+\left(FP+FN\right)/2}, \end{array}$$

where *TP, FP, FN* denote the numbers of true positive, false positive, and false-negative samples, respectively.

#### 2.1.2. Abnormality classifier

The abnormality classifier separates abnormal CXRs from normal ones. It takes as input a PA-view CXR and outputs the probability that the image contains abnormal findings. Only images whose abnormal probabilities are above 0.5 will go to the lesion detector. We trained the abnormality classifier as an EfficientNet-B6 (23) on a dataset of 38,065 PA-view CXRs. All images that were labeled with “No finding” by the radiologists were treated as negative samples, while the rest were considered positive. This model was validated on another dataset of 9,611 images with an F1 score of 0.831. In this study, only the output of the abnormality classifier will be compared to the radiology reports to measure the performance of the whole system.

#### 2.1.3. Lesion detector

The role of the lesion detector is to localize all findings on an abnormal CXR with bounding boxes and, at the same time, classify them into different types of lesions. That is, the system can tell not only whether a CXR is abnormal but also why it is and where the abnormalities come from. An example output of the lesion detector is visualized in Figure 2 where a bounding box of the class “Pulmonary fibrosis” is drawn around the lesion. Out of the 22 local classes in the VinDr-CXR dataset, we only trained the lesion detector on the 17 most prevalent ones as listed in Table 1. The training was performed on 23,524 abnormal CXRs with an EfficientDet-ED4 model (24). The performance of the lesion detector was evaluated on a validation set of 4,470 images using the Average Precision (AP) metric at the Intersection-over-Union (IoU) threshold of 0.4 or shortly AP @ 0.4. This is a standard metric for objection detection models in computer vision (25, 26). Table 1 reports the performance of the lesion detector in terms of AP for each of the 17 classes with an average AP (mAP) of 0.365. However, the model was not validated on the Phu Tho General Hospital data. This is due to the hardness in interpreting the lesion locations in a radiology report.

**Table 1:** Performance of the lesion detector on 17 classes of abnormality.

**Lesion class**	**AP @ 0.4**
Aortic enlargement	0.663
Atelectasis	0.231
Calcification	0.272
Cardiomegaly	0.860
Clavicle fracture	0.459
Consolidation	0.281
Emphysema	0.185
Enlarged PA	0.256
Infiltration	0.318
Interstitial lung disease (ILD)	0.315
Nodule/Mass	0.251
Opacity	0.197
Pleural effusion	0.387
Pleural thickening	0.228
Pneumothorax	0.579
Pulmonary fibrosis	0.340
Rib fracture	0.381
**mAP**	0.365

The bold values are the average performance of all lesson classes.

### 2.2. Integration of AI models to PACS

As shown in Figure 2, the VinDr-CXR system is integrated into the PACS of the hospital through the CXR adapter. This is a web service that pulls all images from the PACS *via* the DICOMweb protocol (27) and only passes CXRs to the AI models of the VinDr-CXR. To check if a scan is a CXR, we rely on the MODALITY and the BODY_PART_EXAMINIED attributes of the DICOM file. In particular, the AI models are triggered only if the value of MODALITY is either CR, DR, or DX and the value of BODY_PART_EXAMINIED is either CHEST or THORAX. These conditions were established by surveying the imaging procedure at the Radiology Department of the Phu Tho General Hospital. For deploying the VinDr-CXR at other clinical sites, the CXR adapter might be slightly modified to catch all CXRs from the PACS.

###  Extraction of radiology reports from HIS

**Figure 3:**
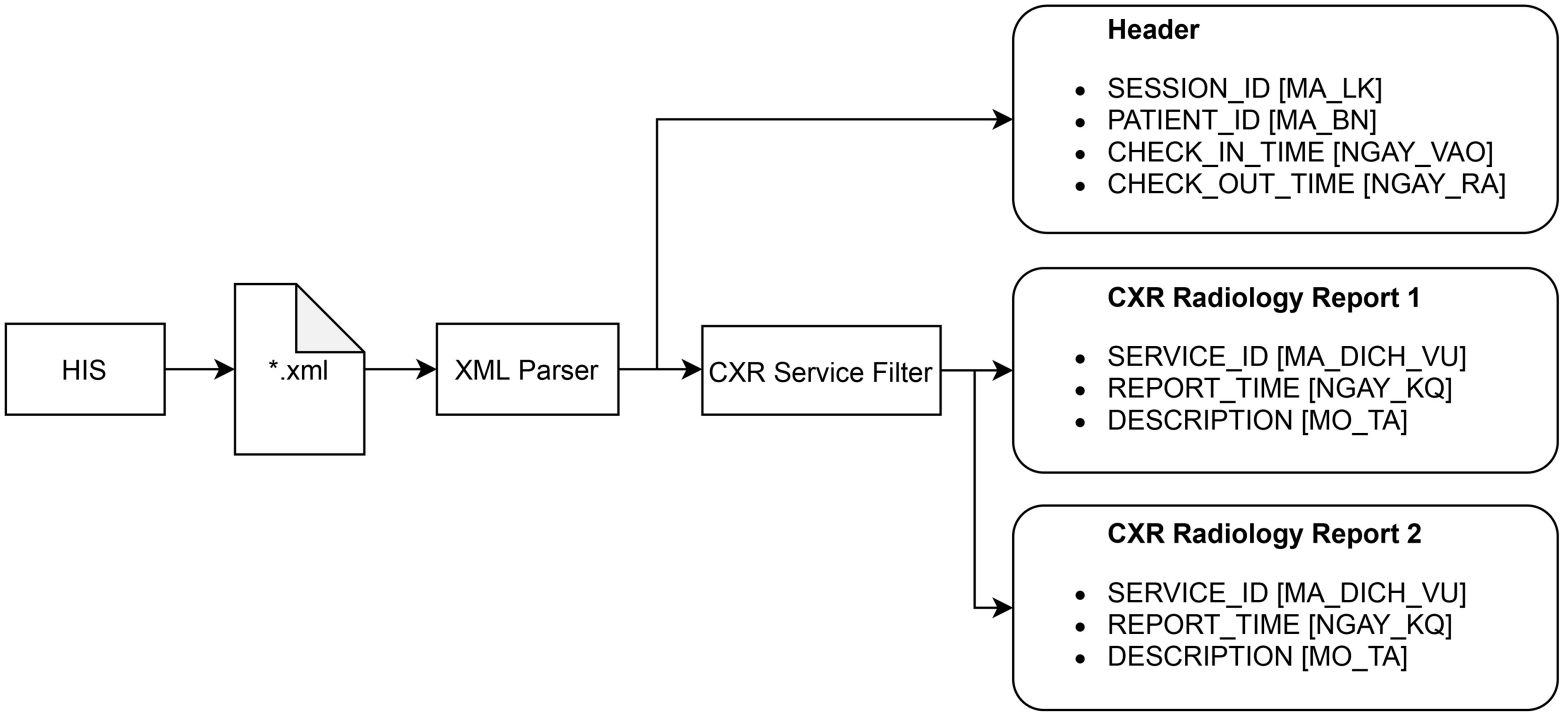
Procedure for extracting all radiology reports of CXR examinations from HIS. The original names of the attributes, which are in Vietnammese, are put inside square brackets.

The radiology reports have to be extracted from HIS according to a procedure described in Figure 3. Each session of examination and treatment is stored in a single Extensible Markup Language (XML) file that can be exported from HIS. A session includes all patient information from the check-in time to the check-out time. The XML parser is used to read all the reports within a session, each of which includes the SERVICE_ID, REPORT_TIME, and DESCRIPTION attributes. The CXR service filter only keeps the reports whose SERVICE_ID matches a fixed value reserved for the CXR imaging by the Vietnamese Ministry of Health. The XML parser can also read the header of a session that includes SESSION_ID, PATIENT_ID, CHECK_IN_TIME, and CHECK_OUT_TIME. These attributes are shared among all radiology reports within the session and will be used, in addition to the REPORT_TIME, to match a radiology report with an AI result of a CXR scan.

### 2.3. Matching AI results with radiology reports

**Figure 4:**
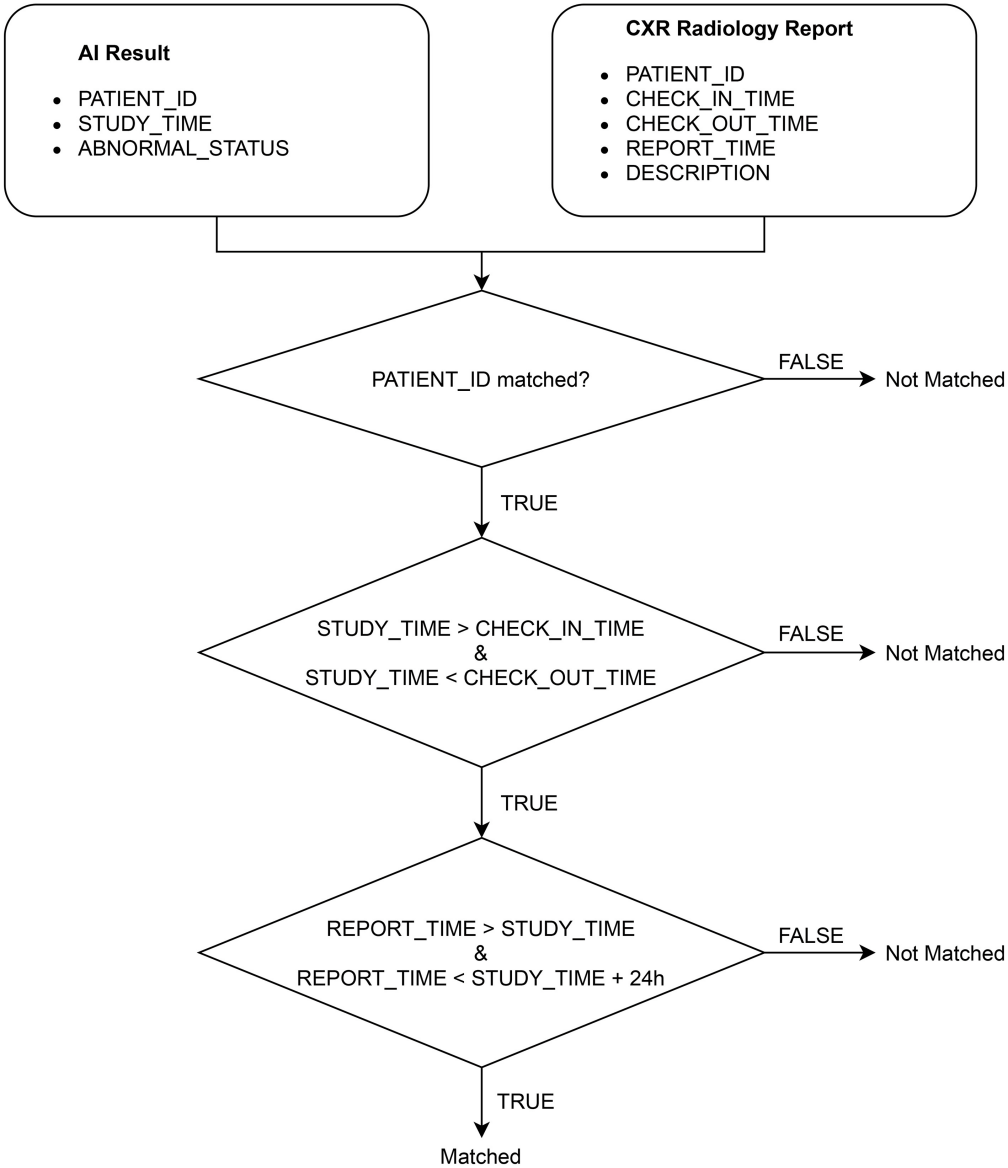
Algorithm for matching an AI result with a radiology report.

After extracting the CXR radiology reports from HIS, we match each of them with an AI result obtained from VinDr-CXR *via* the algorithm illustrated in Figure 4. Note that an AI result includes the ABNORMAL_STATUS (0/1) of a CXR study and is associated with a PATIENT_ID and a STUDY_TIME, which attributes are extracted from the DICOM file. Since HIS and PACS are linked by the PATIENT_ID, the matching algorithm uses this key to check whether an AI result and a radiology report are of the same patient. Next, the STUDY_TIME has to be between the CHECK_IN_TIME and the CHECK_OUT_TIME. Finally, the REPORT_TIME must be within 24 h from the STUDY_TIME, which is a regulated protocol of the hospital. If all the conditions above are satisfied, the AI result and the CXR radiology report are matched.

### 2.4. Comparing AI results to radiology reports

The ABNORMAL_STATUS of an AI result is then compared to the DESCRIPTION of the matched radiology report, if any, to measure the performance of VinDr-CXR in detecting abnormal CXR scans. To that end, we propose a simple template-matching rule to determine if a description is normal. In particular, we observe that a CXR radiology report without any findings always includes four paragraphs that describe the four fixed anatomical regions of the thorax: chest wall, pleura, lung, and mediastinum. The templates for normal descriptions of these four regions are summarized in Table 2. A region is considered normal if one of the corresponding templates exactly appears in the DESCRIPTION of a radiology report. A report is normal if all the four regions are normal. Otherwise, it is abnormal.

**Table 2:** Templates for normal descriptions of the four anatomical regions in a CXR radiology report.

**Anatomical region**	**Templates for normal descriptions**
Chest wall	không thâ`y hình bâ`t thuòng xoung lô´ng nguc
	không thâ`y hình tôn thuòng xuong lô´ng nguc
Pleura	không thâ`y hình tràn dich màng phôi
	không thâ`y hình tràn dich, khí màng phôi
	khôg thâ`y hình tràn khí, tràn dich màng phôi
Lung	nhu mô phôi không thâ`y bâ`t thuòng
Mediastinum	hình tim vâ` trung thâ`t không thâ`y bâ`t thuòng
	hình tim vâ` trung thâ`t bình thuòng

## 3. Results

**Figure 5:**
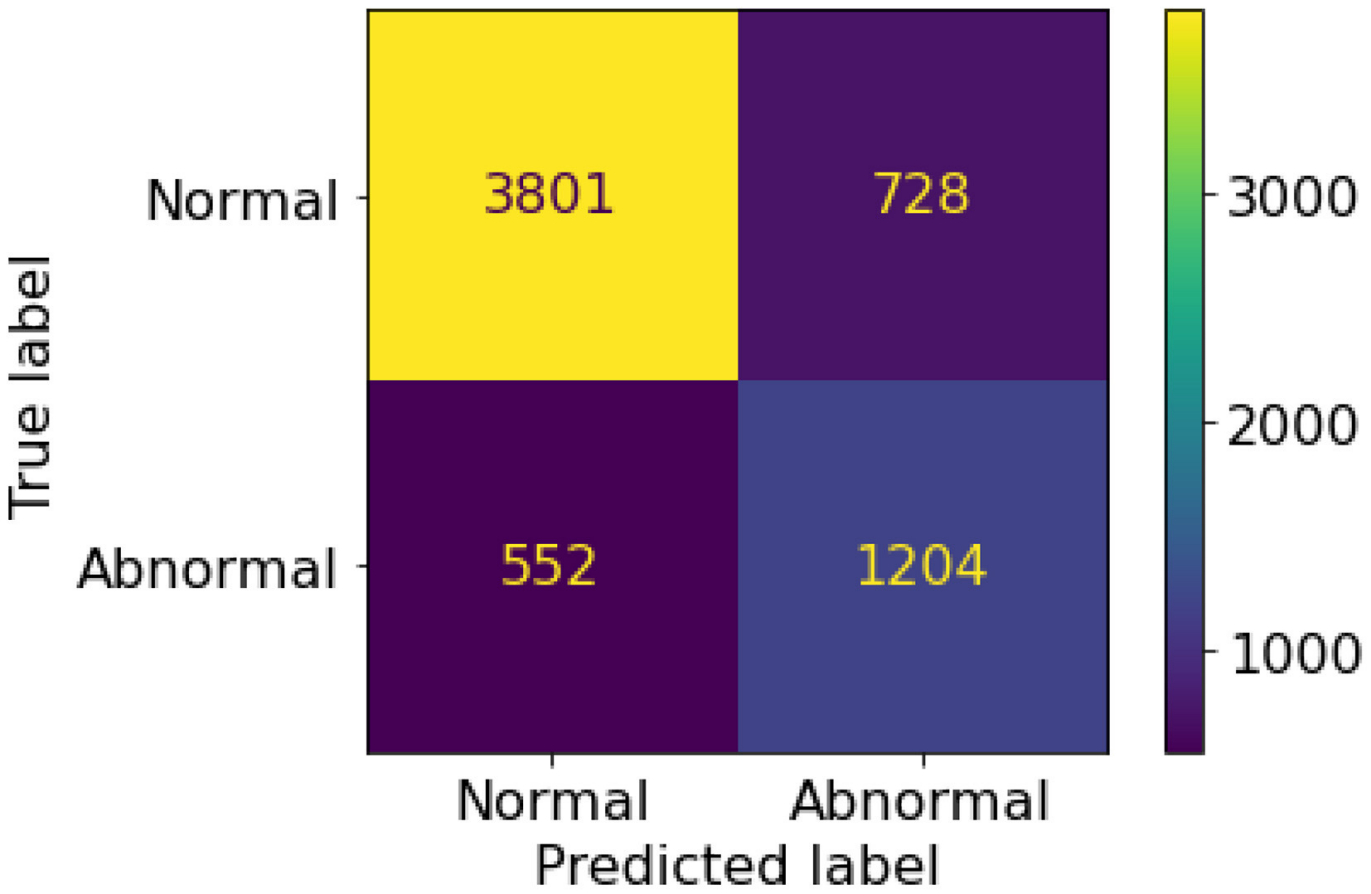
Confusion matrix of the VinDr-CXR abnormality classifier.

**Figure 6:**
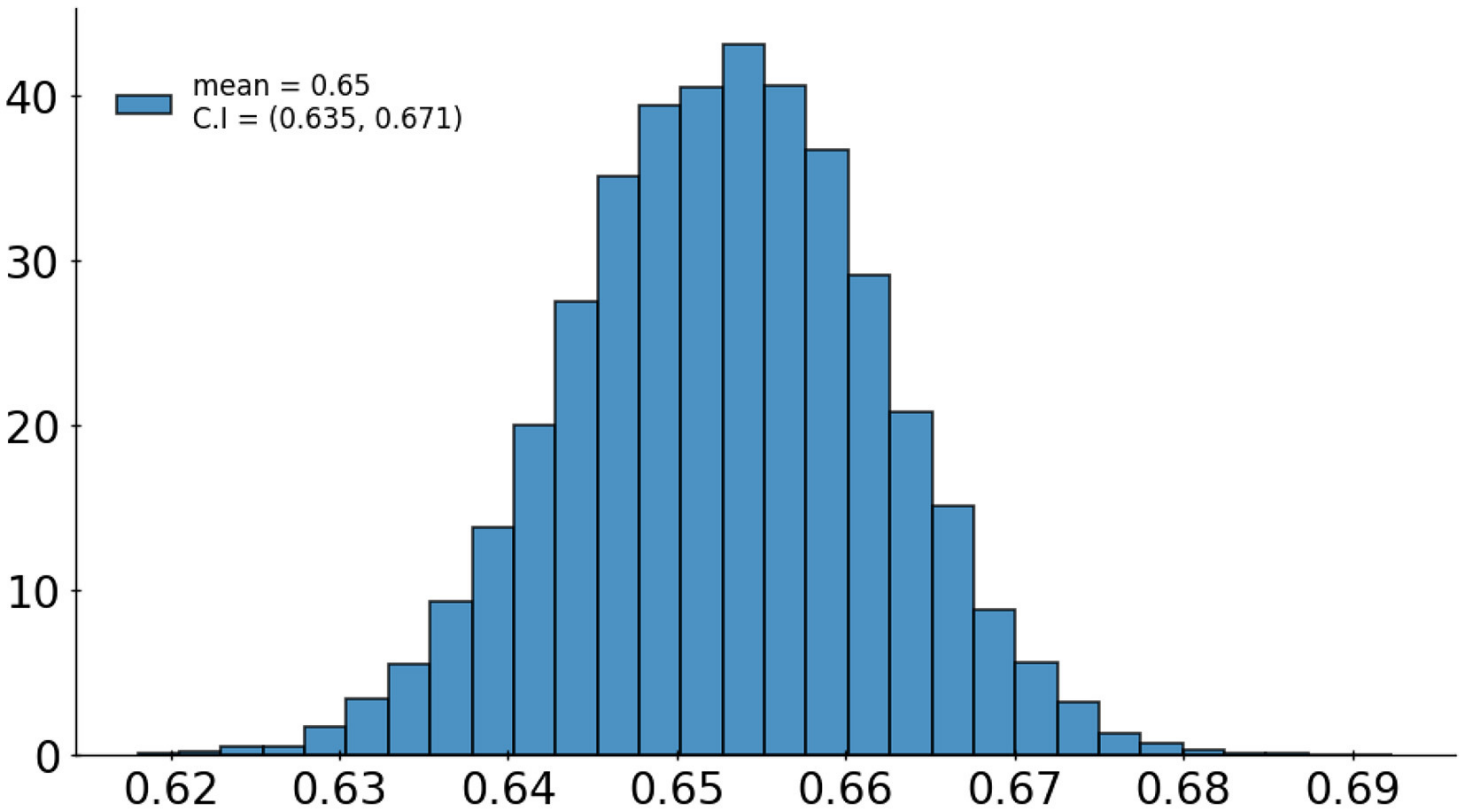
Bootstrap distribution of F1 scores of the VinDr-CXR abnormality classifier over 10,000 samples drawn from 6,285 studies.

A set of 6,585 AI results was obtained by running the VinDr-CXR on all DICOM images in the PACS of the Phu Tho General Hospital throughout November and December of 2020. Meanwhile, another set of 6,687 CXR radiology reports was extracted from the HIS during this period. Applying the matching algorithm to these two sets resulted in 6,285 studies of 5,989 patients with an AI result and a radiology report. By matching the radiology reports of these studies with the templates given in Table 2, we achieved a ground truth of 4,529 (72.1%) normal and 1,756 (27.9%) abnormal cases. The confusion matrix of the VinDr-CXR abnormality classifier over the total 6,285 studies is plotted in Figure 5. This corresponds to an accuracy of 79.6%, a sensitivity of 68.6%, and specificity of 83.9%. We follow (2) to compute the average F1 score on 10,000 bootstrap samples drawn with replacement (21) from the 6,285 studies. We also use the 2.5th and 97.5th percentiles of the bootstrap distribution to establish the 95% confidence interval (CI). The bootstrap distribution of F1 scores is shown in Figure 6, which gives a mean F1 score of 0.653 (95% CI 0.635, 0.671).

## 4. Discussion

It can be seen that the F1 score of our AI system in detecting abnormal CXRs significantly drops from 0.831 to 0.653 when shifting from the training settings to the deployment phase in a clinical site. This might be caused by the shift in the distribution of the CXR images or the additional clinical information received by the radiologists in practice. There are multiple factors that can contribute to the distribution shift such as the varying abnormal/normal ratio between the training set and the test set, the different disease distributions, and the variety of scanners and protocols used at different institutions, etc. For instance, as reported in Section 3, the fraction of abnormal cases in our test set at the deployment site is 27.9% which is significantly different from the similar quantity of 48.4% in the training set. Additionally, our training data contains both CR (Computed Radiography) and DX (Digital Radiography) modalities collected from multiple sources, while all CXR scans at the Phu Tho General Hospital are DX. Although the disease distribution on the test set is not available, we observed an interesting fact that many of the false negative cases were reported with rib/clavicle fractures. This type of errors is likely due to the exceptionally small fraction of fracture lesions in the training set: only 0.46% of the training examples were labeled with rib/clavicle fractures. To have a better understanding of the distribution shift and its effect on the behavior of the AI system, a more thorough error analysis should be carried out by zooming in all the radiology reports.

With an assumption that all the radiology reports at the deployed site are perfectly accurate, the obtained F1 score of 0.653 still exhibits a reasonable level of confidence when deploying the VinDr-CXR in practice. To give a comparison, according to Rajpukar et al. (2), the average performance of 4 radiologists in detecting pneumonia from CXR scans was only 0.387 when measured in F1 score. Of course, there is still room for improving the deploying performance of VinDr-CXR at a particular institution. An obvious way is to fine-tune the system with new data in a continual learning manner. Another way is to increase the generalization ability of the system across institutions by applying more robust data augmentation techniques for medical images (28) during the training phase.

Putting the performance aside, there are still multiple issues with deploying an AI system for radiology in clinical practice. First, the adapter that helps connect the AI pipeline to the hospital's PACS must be able to correctly pick up all CXR scans based on their DICOM tags. This task can be exceptionally hard because values of the DICOM tags are not standardized and often depend on the specific routines of the radiologists and technologists at a particular institution. Second, the PA Classifier, which validates the inputs of VinDr-CXR based on image contents, might also be sensitive to distribution shift and, thus, should be separately evaluated. Third, to protect patient privacy, all DICOM files from a PACS server must be anonymized before being sent to an AI server and all analysis results received from the AI server will then be de-anonymized at the PACS server *via* a lookup table.

One limitation of this study is that it only provides a coarse evaluation of the VinDr-CXR system for classifying a CXR into normal and abnormal categories. To validate the lesion detector of the system, we need a more sophisticated interpretation of the radiology reports that can extract a ground truth comparable to the output of the AI model. Another shortcoming of this work is that the reported clinical validation is for the AI system itself. It is even more critical to assess the effect of such a system on improving the quality of the radiologists' diagnoses. Moreover, an ideal AI-based CAD system should continuously learn from the daily feedback of the doctors rather than staying stationary like our VinDr-CXR. To maximize the benefit of an AI system, a human-machine collaboration paradigm must be carefully designed (29). Finally, the template-based matching algorithm proposed in this paper for identifying a normal/abnormal report only works for hospitals in Vietnam; transferring it to other parts of the world will require further investigations. We plan to address all of these drawbacks in our future research agenda.

## 5. Conclusion

We have discussed in this paper a mechanism for validating the performance of the VinDr-CXR system in classifying normal/abnormal chest radiographs at the Phu Tho General Hospital during the last 2 months of 2020. Once the AI models of the system were trained on an annotated dataset from different sources, they were directly integrated into the PACS of the hospital and never got retrained during the validation period. The performance of the abnormality classifier was prospectively measured by matching and then comparing the obtained AI results with a set of radiology reports exported from the HIS. Since the PACS and the HIS were linked only by patient IDs, we proposed an algorithm to match an AI result of a study with CXR radiology reports. We also adopted a simple template matching rule to decide the abnormal status of a radiology report, which served as a ground-truth reference. We obtained an average F1 score of 0.653 (95% CI 0.635, 0.671) for the abnormality classifier over 10,000 resamples drawn from the 6,285 studies of 5,989. We believe this result has set a significant benchmark for deploying AI systems for chest radiograph analysis in clinical practice.

## Data availability statement

The data analyzed in this study is subject to the following licenses/restrictions: Data cannot be shared publicly due to the risk of violating privacy. Requests to access these datasets should be directed to Dr. Luc Quang Nguyen, Head of Radiology Department, Phu Tho General Hospital; drtranquangluc@gmail.com.

## Ethics statement

The studies involving human participants were reviewed and approved by Institutional Review Board (IRB) of the Phu Tho General Hospital. Written informed consent for participation was not provided by the participants' legal guardians/next of kin because: The need for obtaining informed patient consent was waived because this study did not impact clinical care or workflow at the hospital, and all patient-identifiable information in the data has been removed.

## Author contributions

HN and NHN designed the research. NTN and TN collected and analyzed the data. HN drafted the manuscript. HN, HP, and TN-MN revised the manuscript. All authors have approved the final version.

## Funding

This work was supported by the Vingroup Big Data Institute.

## Conflict of interest

Authors HN, NTN, and TN were employed by the company VinBigData JSC. The remaining authors declare that the research was conducted in the absence of any commercial or financial relationships that could be construed as a potential conflict of interest.

## Publisher's note

All claims expressed in this article are solely those of the authors and do not necessarily represent those of their affiliated organizations, or those of the publisher, the editors and the reviewers. Any product that may be evaluated in this article, or claim that may be made by its manufacturer, is not guaranteed or endorsed by the publisher.
